# Effects of Group‐Based CBT/ERP and ACT on Emotion Dysregulation, Sleep, Anxiety Sensitivity and Psychological Flexibility in Adults With OCD

**DOI:** 10.1002/cpp.70358

**Published:** 2026-11-08

**Authors:** Sara Kerstine Kaya Nielsen, Mette Øllgaard Pedersen, Clas Winding, Kate Wolitzky‐Taylor, Signe Vangkilde, Milan Obaidi, Nicole Rosenberg, Ida Hageman, Martin Balslev Jørgensen, Anne Christine Stuart

**Affiliations:** ^1^ Department of Psychology University of Copenhagen Copenhagen Denmark; ^2^ Mental Health Services Capital Region of Denmark Copenhagen Denmark; ^3^ Department of Psychiatry and Biobehavioral Sciences David Geffen School of Medicine University of California, Los Angeles Los Angeles California USA; ^4^ Mental Health Services Region Zealand Slagelse Denmark

## Abstract

**Introduction:**

Cognitive behavioural therapy with exposure and response prevention (CBT/ERP) is the recommended first‐line psychological treatment for obsessive‐compulsive disorder (OCD). Acceptance and commitment therapy (ACT) has emerged as a promising alternative, yet treatment comparisons have primarily focused on symptom severity and even less is known about their effects on other domains.

**Study Aim:**

We examined the effect of group‐based ACT and CBT/ERP on emotion regulation, anxiety sensitivity, insomnia and psychological flexibility in adults with OCD.

**Methods:**

176 adults with OCD were randomized to CBT/ERP or ACT and assessed with the Difficulties in Emotion Regulation Scale (DERS), Sleep Condition Indicator (SCI), Anxiety Sensitivity Index‐Revised (ASI‐R) and Acceptance and Action Questionnaire for Obsessions and Compulsions (AAQ‐OC) at baseline, post‐treatment, 6‐month and 12‐month follow‐up and analysed with Mixed‐Model Repeated Measures (MMRM).

**Results:**

Both treatments showed similar improvements in the four domains across timepoints.

**Conclusions:**

In line with our hypotheses, our results indicated that ACT may be non‐inferior to CBT/ERP for all secondary outcomes. However, as the trial was powered for the primary outcome (symptom severity) rather than for these secondary measures, our findings should be interpreted as suggestive. Nonetheless, these findings add to the growing evidence that ACT may be considered a viable treatment for OCD.

## Practitioner Points

Group‐based ACT and CBT/ERP produced comparable improvements in emotion regulation difficulties, sleep quality, anxiety sensitivity and psychological flexibility, providing preliminary support for non‐inferiority under the chosen margin across these secondary outcomes. The findings suggest that both ACT and CBT/ERP may have benefits extending beyond reductions in core OCD symptoms, including improvements in broader psychological difficulties that commonly affect patients with OCD. Our results provide additional support for ACT being a viable treatment for OCD.

## 1 Introduction

Obsessive‐compulsive disorder (OCD) is a chronic and potentially disabling mental disorder affecting approximately 1%–3% of the population worldwide (Santomauro et al. 2026). OCD is associated with substantial impairment across multiple areas of life and symptoms often persist without adequate treatment (Öst et al. 2015).

Meta‐analytic evidence shows that cognitive behavioural therapy including exposure and response prevention (CBT/ERP) is an effective treatment for OCD, with large effects relative to waitlist and placebo conditions (Reid et al. 2021). Accordingly, CBT/ERP is recommended as a first‐line psychological treatment in clinical guidelines (NICE 2005). CBT/ERP helps patients systematically confront obsession‐related triggers while refraining from compulsions and other neutralizing behaviours, thereby facilitating new learning and reducing reliance on avoidance and ritualized responses (Foa 2010). Nevertheless, not all patients benefit sufficiently from treatment or complete it. A meta‐analysis reported pooled rates of response and clinically significant change of 65.0% and 50.0%, respectively, for ERP, although definitions varied across studies. Pooled dropout rates were 15.5% for CBT, 19.1% for ERP and 32.0% for ERP/CBT combined with antidepressant medication (Öst et al. 2015). These limitations highlight the need to evaluate alternative treatment approaches.

Acceptance and commitment therapy (ACT) has emerged as a potentially effective treatment for OCD (Evey and Steinman 2023). Rather than primarily targeting the content or frequency of intrusive thoughts, ACT aims to encourage value‐based living, change how individuals relate to internal experiences and strengthen psychological flexibility (Twohig et al. 2006). Although studies indicate that ACT can reduce OCD symptom severity, relatively few trials have compared ACT directly with CBT/ERP (Evey and Steinman 2023). In their systematic review, Evey and Steinman (2023) included 17 studies on ACT, with 8 of them including a control condition and 1 comparing ACT combined with ERP to ERP. Recently, group‐based ACT was found to be non‐inferior to group‐based CBT/ERP in reducing OCD symptoms (Nielsen, Stuart, Winding, Øllgaard, et al. 2025).

Treatment outcomes in studies of CBT/ERP and ACT for OCD have predominantly been evaluated using measures of OCD symptom severity, particularly the Yale–Brown Obsessive Compulsive Scale (Y‐BOCS) (Goodman et al. 1989). Although symptom reduction is a central treatment objective, it may not fully capture changes in psychological functioning and associated clinical difficulties. OCD is indeed also characterized by sleep disturbance, emotion regulation difficulties, anxiety sensitivity and psychological inflexibility. These domains have been associated with the severity or maintenance of OCD (Bluett et al. 2014; Olatunji and Wolitzky‐Taylor 2009; Paterson et al. 2013; See et al. 2022) and may remain clinically relevant even when obsessive‐compulsive symptoms improve. We therefore included sleep disturbance, emotion regulation difficulties, anxiety sensitivity and psychological inflexibility to assess clinically relevant difficulties and theoretically relevant dimensions of functioning that are not fully captured by measures of obsessive‐compulsive symptom severity. Together, they allow us to examine whether the benefits of treatment extend beyond reductions in obsessions and compulsions. Establishing whether these domains change during treatment may also provide a preliminary foundation for subsequent research investigating potential mediators and mechanisms of change (Kazdin 2007).

### 1.1 Anxiety Sensitivity

Several studies have found associations between anxiety sensitivity and OCD symptoms specifically (e.g., Robinson and Freeston 2014). Anxiety sensitivity refers to the tendency to fear anxiety‐related sensations because they are believed to have harmful physical, cognitive, or social consequences (Reiss et al. 1986). Within this expectancy model, such interpretations amplify fear responses to bodily sensations and other anxiety‐related experiences and repeated amplification of fear is thought to maintain anxiety over time (Olatunji and Wolitzky‐Taylor 2009).

Intervention studies have found that both CBT and ACT can reduce anxiety sensitivity (Kraemer et al. 2020; Smits et al. 2008). However, their comparative effects on anxiety sensitivity have not, to our knowledge, been examined specifically among patients with OCD.

### 1.2 Emotion Regulation

Emotion regulation difficulties are consistently associated with OCD symptoms and symptom severity (See et al. 2022). In particular, difficulty tolerating negative emotions and maintaining behavioural control under emotional distress may be relevant to OCD (Nielsen, Stuart, Winding, et al. 2025). Compulsions can temporarily reduce distress, thereby reinforcing avoidance behaviours and limiting opportunities to develop more adaptive responses to unwanted emotions.

CBT has been associated with improved emotion regulation in mixed samples of patients with anxiety‐related disorders (e.g., Asnaani et al. 2020). Nevertheless, comparatively little is known about changes in emotion regulation following CBT/ERP or ACT among patients with OCD, or whether the two treatments differ in their effects on this domain.

### 1.3 Psychological Inflexibility

Psychological inflexibility involves rigid patterns of responding to internal experiences. Cognitive fusion and experiential avoidance represent two central manifestations of psychological inflexibility (Twohig et al. 2006). Cognitive fusion involves responding to thoughts as literal truths rather than as transient mental events, which can result in individuals becoming entangled with and governed by their thoughts. Experiential avoidance refers to attempts to suppress, control, or escape unwanted thoughts, emotions, bodily sensations and memories (e.g., Twohig et al. 2006).

In OCD, cognitive fusion may lead individuals to interpret intrusive thoughts as meaningful indicators of threat or responsibility. Experiential avoidance may subsequently be expressed through compulsions and other neutralizing behaviours intended to reduce the distress experienced by the individual. Both cognitive fusion and experiential avoidance have been associated with greater OCD symptom severity (e.g., Bluett et al. 2014). ACT explicitly targets these two processes and has been shown to increase psychological flexibility among patients with OCD (Evey and Steinman 2023). Improvements in psychological flexibility have also been observed following CBT/ERP (Schubert et al. 2022).

### 1.4 Sleep Quality

The persistent and time‐consuming nature of obsessions and compulsions may interfere with sleep initiation and maintenance. Although sleep disturbances are common across psychiatric disorders, including depression and anxiety disorders, the relationship between OCD and sleep remains comparatively understudied (Paterson et al. 2013).

A large cohort study found that OCD was associated with approximately sevenfold higher odds of insomnia (Sevilla‐Cermeño et al. 2020). Similarly, a review of eight studies found evidence of an elevated prevalence of insomnia among adults with OCD, with commonly reported difficulties including prolonged sleep‐onset latency, disrupted sleep maintenance and reduced sleep quality (Santiago et al. 2025). Taken together, these findings suggest that sleep disturbance represents an important but insufficiently investigated domain of impairment in OCD.

Studies conducted outside the OCD field have also indicated that CBT and ACT may produce comparable improvements in insomnia (e.g., El Rafihi‐Ferreira et al. 2024). However, the comparative effects of ACT and CBT/ERP on sleep difficulties have not, to our knowledge, been examined in an OCD sample.

### 1.5 The Present Study

Although CBT/ERP and ACT are based on distinct theoretical models, both treatments may influence shared psychological processes (Arch and Craske 2008; Fang and Ding 2023; Nielsen et al. 2026). CBT/ERP encourages patients to approach feared situations and internal experiences while refraining from compulsive responses. ACT similarly promotes willingness to experience unwanted thoughts, emotions and bodily sensations without engaging in avoidance, while supporting behaviour guided by personally meaningful values. Both treatments may therefore reduce rigid or avoidant responses to internal distress and increase psychological flexibility (Arch and Craske 2008). This proposition is consistent with comparative evidence suggesting that CBT and ACT may have overlapping effects across symptom‐based and broader psychological outcomes (Fang and Ding 2023).

The present study compared the effects of group‐based ACT and group‐based CBT/ERP on the secondary outcomes sleep, emotion regulation, anxiety sensitivity and psychological inflexibility among adults with OCD from baseline to post‐treatment and through 12‐month follow‐up. We hypothesized that ACT would be non‐inferior to CBT/ERP in improving sleep quality, measured with the Sleep Condition Indicator (SCI); emotion dysregulation, measured with the Difficulties in Emotion Regulation Scale (DERS); anxiety sensitivity, measured with the Anxiety Sensitivity Index–Revised (ASI‐R); and psychological flexibility, measured with the Acceptance and Action Questionnaire for Obsessions and Compulsions (AAQ‐OC).

## 2 Methods

### 2.1 Study Design

The present study is based on data from a multicenter randomized controlled trial comparing group‐based ACT and CBT/ERP for OCD (Nielsen, Stuart, Winding, Øllgaard, et al. 2025). The original trial was registered at ClinicalTrials.gov (ID NCT03852186) and approved by the Danish Research Ethics Committees (ID H‐18046928) and Danish Data Agency (VD‐2018‐376, I‐Suite number: 6626) (Nielsen, Stuart, Winding, Øllgaard, et al. 2025). Participants were recruited from two public outpatient hospital‐based mental health clinics in the Capital Region of Denmark between January 2019 and September 2023. The present study is a secondary analysis of the data from the original trial.

### 2.2 Setting

Participants were referred to the outpatient mental health clinics by general practitioners, hospital departments, or private psychiatrists because of substantial functional impairment related to a mental disorder, complex symptomatology and/or an insufficient response to psychological or pharmacological treatment in primary care.

### 2.3 Participants

Participants were adults aged 18–65 years with a primary diagnosis of OCD according to the Diagnostic and Statistical Manual of Mental Disorders (DSM‐5) criteria. Eligibility required that participants were not receiving psychotherapy at baseline and, if on psychopharmacological treatment, had been on a stable dose of medicine for at least six weeks before study intervention. Participants were required to be fluent in Danish and free of current alcohol or substance use disorder. All referred patients underwent a comprehensive psychiatric examination conducted by a psychiatrist, who assigned a preliminary diagnosis according to ICD–10 criteria. Eligible participants who provided written informed consent were invited to a baseline assessment conducted by the first author. This assessment included clinical interviews including the Y‐BOCS (Goodman et al. 1989) to assess OCD symptom severity and the MINI International Neuropsychiatric Interview (Sheehan et al. 1998) to establish whether participants met DSM–5 criteria for OCD and comorbid psychiatric disorders. The outcome variables used in this study were assessed using self‐reports. Written informed consent was obtained prior to baseline assessment. Self‐report measures were administered via the secure online data capture platform REDCap and post‐treatment assessments were conducted within 14 days of treatment completion (Nielsen, Stuart, Winding, Pedersen, et al. 2025). Sample characteristics are presented in Table 1 and participant flow for the outcomes examined in this study is presented in Figure 1.

**TABLE 1:** Sample characteristics.

	CBT/ERP		ACT	
	(*n* = 65)		(*n* = 89)	
Clinic, *n* (%)				
Clinic 1	26	(40.0)	31	(34.8)
Clinic 2	39	(60.0)	58	(65.2)
Female, *n* (%)	41	(64.1)	66	(74.2)
Age, M (SD)	28.06	(7.12)	28.56	(9.42)
Educational level, *n* (%)				
Lower secondary or less	14	(21.5)	9	(10.1)
Post‐secondary and short‐cycle tertiary	15	(23.1)	23	(25.8)
Bachelor or equivalent	15	(23.1)	23	(25.8)
Master or equivalent	20	(30.8)	30	(33.7)
Employed, *n* (%)	36	(56.3)	43	(48.3)
Yearly income in DKK, median	165,600		150,000	
Have children, *n* (%)	12	(18.8)	17	(19.1)
Religious, *n* (%)	22	(35.5)	25	(28.1)
Previously received a psychiatric diagnosis, *n* (%)	41	(44.1)	52	(58.4)
Personality Inventory for DSM‐5—Brief Form score, M (SD)	22.88	(9.96)	22.09	10.16
Takes psychotropic medication, *n* (%)	30	(46.2)	41	(46.1)
Comorbidity, *n* (%)				
Depression				
Current	6	(10.2)	3	(3.4)
Previous	30	(50.8)	39	(43.8)
Panic disorder	5	(8.5)	4	(4.5)
Agoraphobia	1	(1.7)	1	(1.1)
Social phobia	9	(15.5)	18	(20.2)
Generalized anxiety	0	(0.0)	1	(1.1)
Anorexia nervosa	0	(0.0)	0	(0.0)
Bulimia nervosa	0	(0.0)	2	(2.2)

**FIGURE 1:**
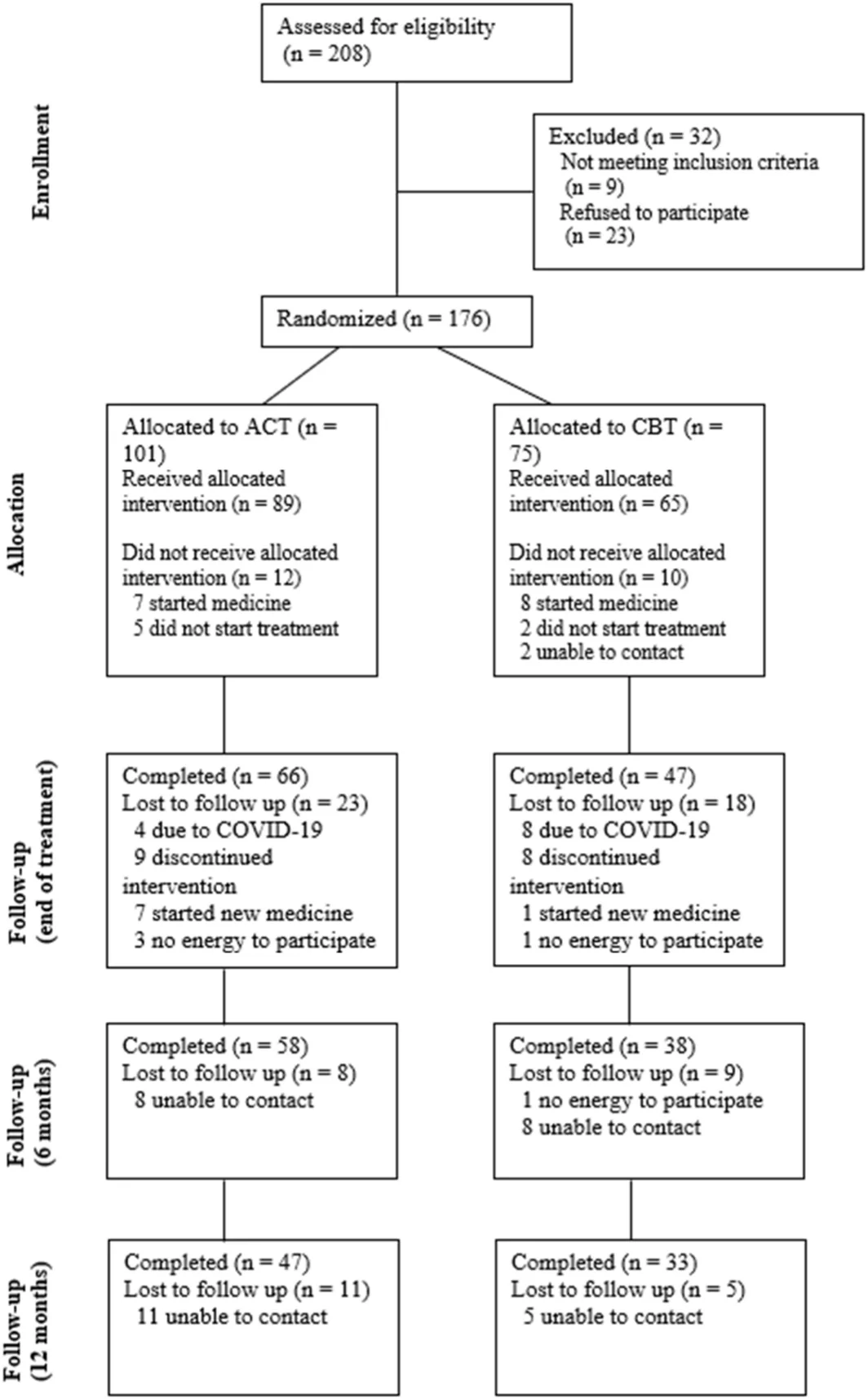
Participant flow.

### 2.4 Randomization and Blinding

Participants were stratified by clinic and randomly allocated to either CBT/ERP or ACT using block randomization with varying block sizes. Allocation concealment was maintained and the randomization sequence was generated using the blockrand package in R; further detail on the randomization procedure is provided in the original trial article (Nielsen, Stuart, Winding, Pedersen, et al. 2025). Participants were informed of their treatment allocation at the first therapy session.

### 2.5 Interventions and Therapists

The intervention consisted of two individual sessions (1 h each) and 14 group sessions (2.5 h each), delivered in groups of up to eight patients (M = 4.41, SD = 1.67, range = 2–7), each led by two therapists. Treatments were delivered by 19 therapists (16 licensed psychologists, one psychiatrist and two psychotherapists), with a mean of 9.4 years of clinical experience at trial onset. Prior to patient inclusion, therapists additionally received 3 days of training in ACT and the ACT treatment manual. All therapists were already trained and experienced in CBT/ERP. Therapists delivered both interventions and received biweekly supervision from experts in ACT (16 years of experience) and CBT/ERP (25 years of experience). As we had to change the format of the interventions due to COVID‐19, we excluded those participants from the analyses (four ACT and eight CBT/ERP).

#### 2.5.1 CBT/ERP

The CBT/ERP protocol, developed by OCD specialists in mental health services and used prior to the original study, focused on cognitive and behavioural strategies targeting dysfunctional thoughts and behaviours associated with OCD. Sessions followed a standardized structure, including review of homework and previous content, introduction of new material and assignment of homework. Core components included psychoeducation on OCD and CBT/ERP, cognitive reappraisal of maladaptive thoughts and beliefs, exposure and response prevention, behavioural experiments and relapse prevention. Exposure exercises were individually tailored and conducted both in session and as homework, with therapist guidance provided individually or in small groups as needed.

#### 2.5.2 ACT

The ACT intervention followed a treatment manual developed by ACT specialists and clinicians specifically for the study, based on previous ACT manuals (Twohig et al. 2010, 2018) and adapted for group therapy. Sessions followed a structured format like the CBT/ERP protocol, including review of homework and previous material, introduction of new content and assignment of homework and behavioural commitment exercises. Core components included psychoeducation on ACT and OCD, identification of personal values and control strategies, enhancement of psychological flexibility using mindfulness, cognitive defusion and acceptance exercises and central ACT interventions (e.g., person in the hole metaphor). Behavioural commitment and value‐based exposure exercises were individually tailored, conducted both in‐session and between sessions and supported by therapists individually or in small groups.

### 2.6 Adherence

Two psychologists assessed manual adherence using study‐specific, session‐specific rating scales informed by procedures described by Wolitzky‐Taylor et al. (2018). Ratings were based on audio recordings of 15% of sessions, sampled across the early, middle and late phases of treatment.

For each prescribed treatment component, raters indicated whether it had been delivered (yes/no). Session adherence was calculated as the number of delivered components divided by the total number of prescribed components, multiplied by 100, for example, the CBT/ERP scale included ratings on homework review, cognitive restructuring, review of maintaining factors, introduction to the exposure record, in vivo exposure and homework planning. The ACT scale included ratings on in‐session mindfulness and present moment exercises including focus on cognitive defusion and acceptance, psychoeducation on self‐as‐context, value‐based step homework, in‐session interviews on values and homework planning.

Adherence was high in both groups, 91.2% for CBT/ERP and 89.7% for ACT (Nielsen, Stuart, Winding, Øllgaard, et al. 2025).

For further details on the sample and interventions, please see Nielsen, Stuart, Winding, Pedersen, et al. (2025).

### 2.7 Participant Flow

Figure 1 presents participant flow and data availability across the four timepoints. The figure shows the number of participants providing observed questionnaire data at baseline, post‐treatment and 6‐ and 12‐month follow‐ups in the CBT/ERP and ACT groups. As shown, the amount of observed data decreased over time and varied across outcome measures.

### 2.8 Measures

#### 2.8.1 The Sleep Condition Indicator (SCI; Espie et al. 2014)

The SCI is an eight‐item self‐report scale measuring concerns about getting to sleep, remaining asleep, sleep quality, daytime personal functioning, daytime performance, duration of sleep problem, nights per week having a sleep problem and extent troubled by poor sleep. Higher scores indicate better sleep quality. The scale has shown robust internal consistency (*α* ≥ 0.86) and showed convergent validity with the Pittsburgh Sleep Quality Index and Insomnia Severity Index (Espie et al. 2014). In our sample, the internal consistency was similarly good (*α*_baseline_ = 0.86, *α*_post_ = 0.89, *α*_6‐FU_ = 0.86, *α*_12‐FU_ = 0.87).

#### 2.8.2 Difficulties in Emotion Regulation Scale (DERS; Gratz and Roemer 2004)

The DERS is a self‐report measure with 36 items that assess to what extent difficult emotions are impacting daily life. Scored on a 5‐point Likert scale, the DERS yields a total score of difficulties in emotion regulation, along with six specific subscales. Higher scores indicate more difficulties in emotional regulation. In this study, we included the total score, which demonstrated good‐to‐excellent internal consistency (*α*_baseline_ = 0.91, *α*_post_ = 0.88, *α*_6‐FU_ = 0.88, *α*_12‐FU_ = 0.89).

#### 2.8.3 Acceptance and Action Questionnaire for Obsessions and Compulsions (AAQ‐OC; Jacoby et al. 2018)

The AAQ‐OC is a 13‐item self‐report measure of experiential avoidance in response to unwanted intrusive thoughts, scored on a 7‐point Likert scale. It comprises the two subscales, Valued Action and Willingness, which can be summed into a total score (Jacoby et al. 2018). Higher scores indicate more avoidance and poorer acceptance. In the original validation sample, the total score and the Valued Action and Willingness subscales showed good internal consistency (Cronbach's *α* = 0.93, 0.92 and 0.82, respectively; Jacoby et al. 2018). In the present study, we only used the total score, which showed good‐to‐excellent internal consistency (*α*_baseline_ = 0.85, *α*_post_ = 0.89, *α*_6‐FU_ = 0.91, *α*_12‐FU_ = 0.90).

#### 2.8.4 Anxiety Sensitivity Index‐Revised (ASI‐R; Deacon et al. 2003)

ASI‐R assesses beliefs that anxiety‐related bodily sensations (e.g., rapid heartbeat, dizziness) are harmful, with sensations categorized as having either negative social, physical, or mental consequences. This is assessed via four subscales corresponding to: (i) fear of respiratory symptoms, (ii) fear of publicly observable anxiety reactions, (iii) fear of cardiovascular symptoms and (iv) fear of cognitive dyscontrol. These can be summed into a total score, which was used in the present study. Higher scores indicate greater anxiety sensitivity. In our sample, the total score showed excellent internal consistency (*α*_baseline_ = 0.96, *α*_post_ = 0.97, *α*_6‐FU_ = 0.95, *α*_12‐FU_ = 0.97).

### 2.9 Statistical Analysis

All analyses were performed using the lme4 package (Bates et al. 2015) in R v. 4.3.2 and evaluated at a significance level of 5% (two‐tailed). Missing data were handled by multivariate imputation using chained equations (van Buuren and Groothuis‐Oudshoorn 2011). Separate imputations were performed in the two RCT groups.

All hypotheses were tested under the intention‐to‐treat principle using repeated linear mixed models to account for the correlation induced by the participants attending the same group in therapy. The dependent variables sleep quality, psychological inflexibility, anxiety sensitivity and emotion dysregulation were tested using four separate models. The within‐subjects level was the four timepoints with the baseline assessment as the reference group. The between‐subjects level was the two treatment groups. Covariates included psychotropic medication (selective serotonin reuptake inhibitor yes/no) and personality disorder traits measured as the total score on the Personality Inventory for DSM‐5—Brief Form (Krueger et al. 2012).

A non‐inferiority approach was used to interpret the differences in the outcomes for ACT and CBT/ERP groups. As scale‐specific minimal clinically important differences do not exist, to the best of our knowledge, the non‐inferiority margin was defined using a distribution‐based approach. As per recommendations, we used 0.5 standard deviation for the total sample scores at baseline as the margin (Leichsenring et al. 2018). On this basis, the non‐inferiority margins were set at 3.61 for sleep quality (SCI), 5.74 for psychological inflexibility (AAQ‐OC), 15.70 for anxiety sensitivity (ASI‐R) and 11.94 for emotion dysregulation (DERS) (see Table 2 for descriptive statistics).

**TABLE 2:** Descriptive statistics of the outcome variables.

	ACT			CBT/ERP			Total		
	M	SD	Range	M	SD	Range	M	SD	Range
SCI									
Baseline	18.90	7.10	5–32	17.81	7.37	1–31	18.43	7.21	1–32
Post	19.74	6.44	1–32	18.73	8.66	2–32	19.32	7.42	1–32
6‐month follow‐up	21.52	6.75	5–32	21.28	7.18	7–32	21.42	6.89	5–32
12‐month follow‐up	20.18	7.44	3–30	22.42	6.78	9–32	20.93	7.24	3–32
AAQ‐OC									
Baseline	67.26	11.68	38–91	66.80	11.28	36–91	67.08	11.47	36–91
Post	55.78	13.78	16–81	54.30	12.06	28–85	55.17	13.05	16–85
6‐month follow‐up	53.26	13.90	17–85	49.42	14.55	16–87	51.71	14.19	16–87
12‐month follow‐up	50.81	15.00	18–86	51.94	11.26	29–78	51.15	13.88	18–86
ASI‐R									
Baseline	41.87	29.33	1–131	41.20	34.15	2–126	41.58	31.40	1–131
Post	35.14	29.68	0–117	35.81	29.98	0–127	35.42	29.66	0–127
6‐month follow‐up	37.41	27.18	0–109	26.31	19.47	0–70	33.12	24.95	0–109
12‐month follow‐up	31.14	27.84	0–116	21.56	23.07	0–92	28.19	26.61	0–116
DERS									
Baseline	93.06	23.13	49–147	97.10	24.89	46–149	94.75	23.88	46–149
Post	88.03	18.90	51–133	88.40	22.72	47–139	88.18	20.41	47–139
6‐month follow‐up	83.62	18.24	54–122	82.92	21.78	52–124	83.37	19.46	52–124
12‐month follow‐up	78.62	19.48	50–122	73.56	18.16	49–113	77.00	19.03	49–122
Y‐BOCS									
Baseline	22.34	5.71	7–35	23.44	4.91	11–33	22.83	5.38	7–35
Post	16.25	6.09	1–31	16.86	6.55	1–31	16.50	6.26	1–31
6‐month follow‐up	14.23	7.65	0–29	14.68	7.05	0–27	14.41	7.38	0–29
12‐month follow‐up	13.43	7.12	0–29	14.71	8.87	0–29	13.89	7.75	0–29

Abbreviations: AAQ‐OC = Acceptance and Action Questionnaire for Obsessions and Compulsive; ASI‐R = Anxiety Sensitivity Index‐Revised; DERS = Difficulties in Emotion Regulation Scale; SCI = Sleep Condition Indicator; Y‐BOCS = Yale‐Brown Obsessive Compulsion Scale.

Given the fact that this study explored the effect of ACT and CBT/ERP on secondary outcomes, we did not adjust for multiple comparisons.

## 3 Results

Descriptive statistics are presented in Table 2. Overall, psychological inflexibility (AAQ‐OC), anxiety sensitivity (ASI‐R) and emotion dysregulation (DERS) scores decreased over the four timepoints in both groups, whereas they increased for sleep quality (SCI).

The results are presented in Table 3. For both sleep quality (SCI) and anxiety sensitivity (ASI‐R), we saw no significant differences in scores from baseline to post. However, the participants' sleep quality (SCI) significantly increased and anxiety sensitivity (ASI‐R) significantly decreased for the two follow‐up assessments. For both psychological inflexibility (AAQ‐OC) and emotion dysregulation (DERS), the scores significantly decreased at all three timepoints compared to baseline. There were no significant interaction terms between RCT group and timepoint (all *p*s ≥ 0.127), suggesting that these improvements were similar in the two groups.

**TABLE 3:** Results of the primary analyses (CBT/ERP as the reference group).

	b	p	95% CI
Group differences in SCI	0.94	0.420	[−1.36; 3.25]
Post vs. baseline	0.58	0.530	[−1.24; 2.41]
6‐month follow‐up vs. baseline	3.03	< 0.001	[0.96; 5.09]
12‐month follow‐up vs. baseline	3.17	0.001	[0.67; 5.67]
Group differences in AAQ‐OC	0.40	0.848	[−3.74; 4.55]
Post vs. baseline	−12.29	< 0.001	[−15.76; −8.83]
6‐month follow‐up vs. baseline	−18.25	< 0.001	[−22.19; −14.30]
12‐month follow‐up vs. baseline	−14.33	< 0.001	[−19.39; −9.26]
Group differences in ASI‐R	1.07	0.822	[−8.30; 10.44]
Post vs. baseline	−4.05	0.186	[−10.07; 1.97]
6‐month follow‐up vs. baseline	−12.46	< 0.001	[−19.46; −5.46]
12‐month follow‐up vs. baseline	−9.11	0.004	[−17.86; −0.36]
Group differences in DERS	−2.92	0.369	[−9.31; 3.46]
Post vs. baseline	−6.72	< 0.001	[−11.75; −1.69]
6‐month follow‐up vs. baseline	−10.31	< 0.001	[−16.31; −4.32]
12‐month follow‐up vs. baseline	−14.51	< 0.001	[−21.68; −7.33]

Abbreviations: AAQ‐OC = Acceptance and Action Questionnaire for Obsessions and Compulsions; ASI‐R = Anxiety Sensitivity Index‐Revised; DERS = Difficulties in Emotion Regulation Scale; SCI = Sleep Condition Indicator.

As seen in Table 3 and Figure 2, there were no significant group differences for any of the measures. However, the 95% CI was below the non‐inferiority margins for all the outcomes, suggesting that ACT was non‐inferior to CBT/ERP treatment. Group contrasts for each of the timepoints are presented in the Supporting Information. The 95% CI was only below the data‐driven non‐inferiority margin at baseline for psychological inflexibility (AAQ‐OC) and anxiety sensitivity (ASI‐R) measures.

**FIGURE 2:**
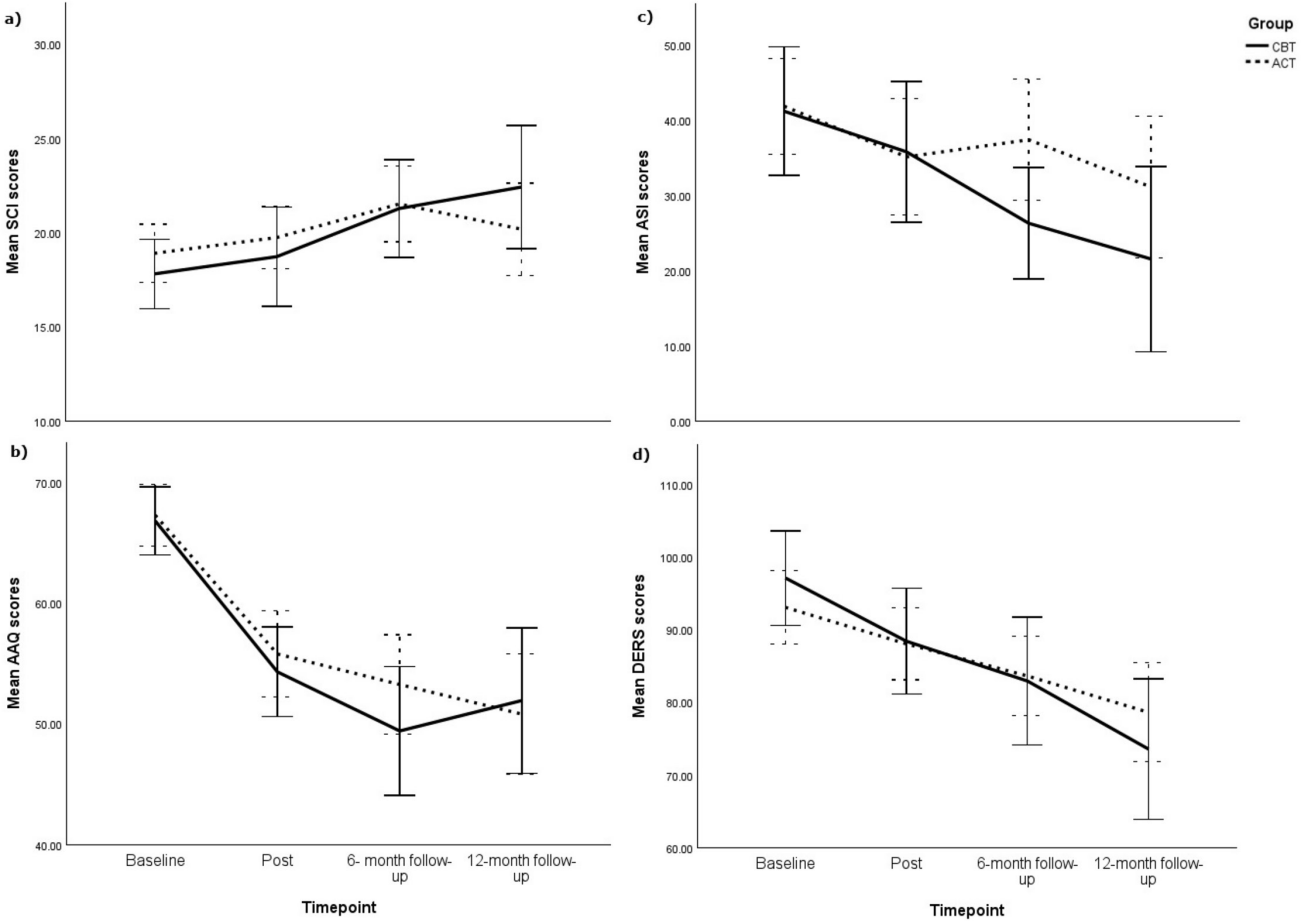
(a) Mean scores of SCI, (b) AAQ‐OC, (c) ASI‐R and (d) DERS for the two interventions at the four timepoints. Error bars are 95% CI.

## 4 Discussion

The present study examined the effects of group‐based CBT/ERP and ACT on the secondary outcomes emotion dysregulation, sleep quality, anxiety sensitivity and psychological inflexibility. Consistent with our hypotheses, no statistically significant between‐group differences were found, suggesting that ACT was non‐inferior to CBT/ERP across all outcomes according to the chosen margins. However, group contrasts at the four different assessment points only suggested non‐inferiority at baseline for psychological inflexibility and anxiety sensitivity (see Supporting Information Table S1). These findings may be attributable to the substantial attrition at follow‐up and our results should thus be interpreted cautiously. Overall, the two treatments showed comparable and sustained improvements.

Despite their distinct theoretical frameworks and therapeutic strategies, CBT/ERP and ACT both involve sustained contact with distressing internal experiences without engaging in habitual avoidance or control strategies. A possible explanation for the possible similarities may therefore reflect shared therapeutic processes rather than treatment‐specific mechanisms. CBT/ERP primarily seeks to modify threat‐related beliefs and disrupt the reinforcement of compulsions through repeated exposure and response prevention (Foa 2010). ACT, by contrast, explicitly targets psychological inflexibility by promoting acceptance, cognitive defusion, mindfulness and value‐based action (Twohig et al. 2006). In practice, however, both treatments require patients to remain in contact with distressing thoughts, emotions and bodily sensations without engaging in habitual avoidance or control strategies. Both may therefore increase willingness to experience distress, weaken avoidance‐based responding and promote greater psychological flexibility.

These possible shared processes provide a coherent explanation for the possibly comparable changes in emotion dysregulation, sleep quality, anxiety sensitivity and psychological inflexibility observed in the present study. Rather than representing entirely separate treatment targets, these variables may reflect interrelated aspects of a broader shift in how patients respond to unwanted internal experiences (Arch and Craske 2008). Learning to experience distress without compulsive regulation may be associated with improved emotion regulation, reduced catastrophic responses to anxiety‐related sensations and increased psychological flexibility. By the same logic, reduced arousal, rumination and attempts to control internal experiences may contribute to improved sleep. Thus, although CBT/ERP and ACT conceptualize therapeutic change differently, both may help patients develop less avoidant and more adaptive responses to distress and improve sleep.

Previous research broadly supports our results, although the evidence specific to OCD remains limited, making direct comparisons difficult. Reductions in emotion regulation difficulties have been reported following both CBT/ERP and ACT (Asnaani et al. 2020; Fang and Ding 2023). Arch, Eifert, et al. (2012) found comparable reductions in anxiety sensitivity following CBT and ACT. Furthermore, psychological inflexibility has been found to decrease following CBT for OCD, even though it is not an explicit treatment target (Schubert et al. 2022). Studies directly comparing CBT/ERP and ACT for OCD have similarly found improvements in psychological flexibility following both treatments (Twohig et al. 2018). Although some findings suggest that specific processes may be more strongly associated with one treatment—for example, greater short‐term improvements in psychological inflexibility following ACT or a stronger relationship between anxiety sensitivity and outcomes following CBT—the evidence does not indicate a consistent differentiation between the treatments (e.g., Arch, Wolitzky‐Taylor, et al. 2012).

Importantly, the observed changes cannot be interpreted as evidence that these variables constitute mechanisms of change. The aim of our study was not to investigate mechanisms of change and the analyses did not establish whether changes in emotion regulation, sleep quality, anxiety sensitivity, or psychological flexibility preceded subsequent reductions in OCD symptoms. Future studies using repeated assessments during treatment and temporally sensitive mediation analyses are needed to determine whether these processes contribute to symptom improvement or instead change as a consequence of it. Evidence that changes in these variables precede symptom reduction in both treatments would support the possible shared functional pathways through which theoretically distinct interventions may produce comparable clinical outcomes.

### 4.1 Clinical Implications

CBT/ERP is recommended as a first‐line psychological treatment for OCD based on consistent evidence from RCTs and meta‐analyses (NICE 2005; Öst et al. 2015). By directly comparing CBT/ERP and ACT and identifying broadly similar patterns of change across emotion dysregulation, sleep quality, anxiety sensitivity and psychological flexibility, the present study adds to the evidence supporting ACT as a potentially viable intervention for OCD. When more than one treatment is empirically supported, treatment selection may more fully incorporate patient preferences, individual clinical characteristics, therapist expertise and clinical judgement, consistent with the principles of evidence‐based practice (Cook et al. 2017). The findings may therefore support greater flexibility in treatment selection.

### 4.2 Strengths and Limitations

The present study reports secondary analyses of data from an RCT, providing a rigorous basis for comparing changes following CBT/ERP and ACT. Its longitudinal design, with assessments conducted at baseline, post‐treatment and 6‐ and 12‐month follow‐ups, enabled examination of both immediate and sustained changes.

Several limitations should nevertheless be considered. First, the original trial was powered to detect effects on OCD symptom severity measured with the Y‐BOCS. The sample size was therefore not determined specifically for the four measures used in this study. This may have reduced the precision of estimates for these secondary outcomes and limited the ability to detect small between‐group differences, as reflected in the wide confidence intervals. In line with this, the outcome variables in this article were not registered as part of the original protocol.

Second, no empirically established minimal clinically important differences were available for the four outcomes. The non‐inferiority margins were therefore defined using a distribution‐based approach. Although such approaches are frequently used when established clinical thresholds are unavailable (Leichsenring et al. 2018; Mouelhi et al. 2020), it remains uncertain whether a margin of 0.5 SD represents a clinically meaningful difference for each construct. Future studies should seek to establish anchor‐based thresholds that reflect changes considered meaningful by patients and clinicians.

Third, the exclusive use of self‐report measures may limit construct validity. Incorporating behavioural, clinician‐rated, physiological, or digital measures could provide a more comprehensive assessment of change.

Fourth, missing follow‐up data may have affected the long‐term estimates. Missing data imputation was conducted and mixed models for repeated measures use all available observations and can provide valid estimates under the assumption that data are missing at random. However, this assumption cannot be verified using the observed data alone and missingness may have depended on unmeasured characteristics or outcomes. Systematic attrition could therefore have biased the follow‐up estimates.

Fifth, although treatment adherence was rated and found to be high, treatment quality was not formally assessed. All therapists participated in biweekly supervision based on audio recordings of treatment sessions; however, supervision does not substitute for systematic ratings of treatment quality. Consequently, we could not establish whether treatment quality was comparable across ACT and CBT/ERP or examine whether variation in therapist competence contributed to differences in outcomes.

Last, data on additional psychological or pharmacological treatment received during the follow‐up period were not collected. Consequently, we could not account for subsequent treatment that may have influenced secondary outcomes.

### 4.3 Conclusion and Future Directions

CBT/ERP and ACT were associated with comparable improvements in emotion dysregulation, sleep quality, anxiety sensitivity and psychological flexibility. These findings contribute to the growing evidence that group‐based ACT may represent a viable treatment option for OCD.

Future research should examine whether changes in these processes precede and predict subsequent reductions in OCD symptoms and therefore can be conceptualized as shared mechanisms of change. As noted above, repeated within‐treatment assessments and mediation analyses are needed to clarify these mechanisms. Research should also investigate whether particular processes are more important in one treatment than the other and whether individual baseline characteristics predict differential process–outcome relationships.

If changes in emotion dysregulation, anxiety sensitivity, psychological flexibility, or sleep quality are shown to precede subsequent symptom improvement in both CBT/ERP and ACT, this would support the presence of shared functional pathways across the treatments. Such findings would not necessarily eliminate the theoretical distinctions between the approaches, but they could clarify how different therapeutic strategies converge on common processes that contribute to recovery from OCD.

## Funding

This study was funded by unrestricted grants from the Lundbeck Foundation (ID: R264‐2017‐2034).

## Conflicts of Interest

The authors declare no conflicts of interest.

## Supporting information




**Table S1:** Group contrasts at each timepoint (CBT/ERP is the reference group).

## Data Availability

Data cannot be shared publicly because our dataset includes subjects who did not explicitly consent to the publication of their pseudonymized data, which is required to comply with the Danish Data Protection Act. Therefore, publishing the dataset does not align with the ethical standards of data management and regulations upheld at the University of Copenhagen. Data are available from the Data Management and Information Security department at the University of Copenhagen.
