# Supplementary material for: Effects of Group‐Based CBT/ERP and ACT on Emotion Dysregulation, Sleep, Anxiety Sensitivity and Psychological Flexibility in Adults With OCD
Source: Clin Psychol Psychother. 2026 Nov 8;33(6):e70358. doi: 10.1002/cpp.70358 (PMC13745865; doi:10.1002/cpp.70358)
Supplement: Supplementary file 1 [file CPP-33-e70358-s001.docx]

| **Supplementary Table S1.** Group contrasts at each timepoint (CBT/ERP is the reference group) | | | | | | | | | |  |  |  |  |  |  |
| --- | --- | --- | --- | --- | --- | --- | --- | --- | --- | --- | --- | --- | --- | --- | --- |
|  | Baseline | | |  | Post | | |  | 6-month follow-up | | |  | 12-month follow-up | | |
|  | *b* | *p* | 95% CI |  | *b* | *p* | 95% CI |  | *b* | *p* | 95% CI |  | *b* | *p* | 95% CI |
| SCI | 8.92 | .732 | [-49.3; 67.2] |  | -7.46 | .677 | [-44.0; 29.1] |  | -7.51 | .690 | [-46.4; 31.3] |  | -9.36 | .680 | [-58.3; 39.6] |
| AAQ-OC | -0.04 | .987 | [-4.55; 4.48] |  | -0.68 | .801 | [-6.09; 4.74] |  | -1.39 | .667 | [-8.11; 5.34] |  | 0.30 | .919 | [-5.64; 6.24] |
| ASI-R | 1.14 | .856 | [-11.2; 13.5] |  | 4.56 | .630 | [-15.9; 25.1] |  | 0.31 | .979 | [-26.3; 26.9] |  | 7.02 | .653 | [-30.6; 44.6] |
| DERS | 7.70 | .762 | [-45.1; 60.5] |  | -11.6 | .682 | [-71.9; 48.7] |  | -19.4 | .618 | [-108.1; 69.2] |  | -25.1 | .754 | [-233.7; 183.5] |
